# Bortezomib Sustains T Cell Function by Inducing miR-155-Mediated Downregulation of SOCS1 and SHIP1

**DOI:** 10.3389/fimmu.2021.607044

**Published:** 2021-02-25

**Authors:** Ariana N. Renrick, Menaka C. Thounaojam, Maria Teresa P. de Aquino, Evan Chaudhuri, Jui Pandhare, Chandravanu Dash, Anil Shanker

**Affiliations:** ^1^ Department of Microbiology, Immunology and Physiology, School of Medicine, Meharry Medical College, Nashville, TN, United States; ^2^ School of Graduate Studies and Research, Meharry Medical College, Nashville, TN, United States; ^3^ Department of Biochemistry, Cancer Biology, Neuroscience and Pharmacology, School of Medicine, Meharry Medical College, Nashville, TN, United States; ^4^ Center for AIDS Health Disparities Research, Meharry Medical College, Nashville, TN, United States; ^5^ Vanderbilt Institute for Infection, Immunology and Inflammation, Vanderbilt University, Nashville, TN, United States; ^6^ Host-Tumor Interactions Research Program, Vanderbilt-Ingram Cancer Center, Vanderbilt University, Nashville, TN, United States

**Keywords:** immunomodulators, lymphocyte function, cancer immunotherapy, microRNA, immunosuppression, tumor microenvironment, T cell exhaustion, antitumor immunity

## Abstract

Suppressive mechanisms operating within T cells are linked to immune dysfunction in the tumor microenvironment. We have previously reported using adoptive T cell immunotherapy models that tumor–bearing mice treated with a regimen of proteasome inhibitor, bortezomib - a dipeptidyl boronate, show increased antitumor lymphocyte effector function and survival. Here, we identify a mechanism for the improved antitumor CD8^+^ T cell function following bortezomib treatment. Intravenous administration of bortezomib at a low dose (1 mg/kg body weight) in wild-type or tumor-bearing mice altered the expression of a number of miRNAs in CD8^+^ T cells. Specifically, the effect of bortezomib was prominent on miR-155 - a key cellular miRNA involved in T cell function. Importantly, bortezomib–induced upregulation of miR-155 was associated with the downregulation of its targets, the suppressor of cytokine signaling 1 (SOCS1) and inositol polyphosphate-5-phosphatase (SHIP1). Genetic and biochemical analysis confirmed a functional link between miR-155 and these targets. Moreover, activated CD8^+^ T cells treated with bortezomib exhibited a significant reduction in programmed cell death-1 (PD-1) expressing SHIP1^+^ phenotype. These data underscore a mechanism of action by which bortezomib induces miR-155–dependent downregulation of SOCS1 and SHIP1 negative regulatory proteins, leading to a suppressed PD-1–mediated T cell exhaustion. Collectively, data provide novel molecular insights into bortezomib–mediated lymphocyte–stimulatory effects that could overcome immunosuppressive actions of tumor on antitumor T cell functions. The findings support the approach that bortezomib combined with other immunotherapies would lead to improved therapeutic outcomes by overcoming T cell exhaustion in the tumor microenvironment.

## Introduction

Disruption of immune regulatory networks takes place in the tumor microenvironment. Intrinsic suppressive mechanisms of T cells are linked to immune dysfunction and cancer progression (1). This is evident by the clinical efficacy of immune-based therapies targeting the inhibitory immune checkpoint molecules on T cells (2). However, current immunotherapies are ineffective at inducing durable responses in a majority of patients and cancer types, and we have yet to fully grasp how we can intersect the inhibitory mechanisms during the immune responses to cancer (2, 3). There is, thus, a critical need to identify novel approaches that can abrogate suppressive mechanisms operating within T cells to develop highly effective immunotherapy.

Bortezomib (Velcade™/PS-341) is a synthetic dipeptidyl boronate proteasome inhibitor that has been approved by the U.S. Food and Drug Administration for the treatment of multiple myeloma (4, 5) and mantle cell lymphoma (6, 7). Studies from our laboratory have demonstrated that bortezomib also sensitizes mouse and human solid tumor cells to apoptosis by upregulating caspase-8 activity in the death-inducing signaling complex following death receptor ligation on tumor cells (8, 9). Subsequently, the use of bortezomib was extended to relapsed or refractory myeloma (10) and advanced stage non–small-cell lung cancer (11). Recently, we reported that bortezomib treatment in mice bearing solid tumors influenced tumor microenvironment by increasing the levels of immunostimulatory cytokines IL-2, IL-12 and IL-15 (12). It also enhanced the production of IFN*γ* and expression of effector molecules perforin, granzyme B, *eomesodermin* and FasL in CD8^+^ T cells (13, 14). These bortezomib-mediated immune effects significantly improved adoptive T cell therapy against adenocarcinomas in mice by predominantly enhancing FasL–mediated CD8^+^ T cell cytotoxicity and tumor-free survival (14). However, the mechanism by which bortezomib modulates these T cell intrinsic effects culminating in an increased antitumor effector function remains unclear.

MicroRNAs (miRNA) are short noncoding RNAs that regulate post-transcriptional expression of proteins involved in various biological processes, including immune function (15). They regulate degradation and/or translational repression of mRNAs, which contain complementary sequences to that of the miRNA (16, 17). Emerging evidence show that miRNAs are involved both in the adaptive and innate immune responses. For example, miR-181 regulates mature B cell differentiation and early B cell development. Similarly, monocyte differentiation is linked to miR-17 through 92 family of miRNA clusters (18–21). Moreover, Toll like receptor (TLR) signaling induces miR-155 expression along with miR-146a and miR-21 (22–28). Additionally, miR-155 is required for effector CD8^+^ T cell responses against viral infection and cancer (29, 30). For instance, CD8^+^ T cells from miR-155^-/-^ mice exhibit decreased IFN*γ* mRNA expression (29). Lower amounts of IFN*γ* ^+^ T cells are also reported among the tumor-infiltrating lymphocytes (31). This was supported by dysfunctional antitumor immunity in T cell-specific miR-155^-/-^ mice (32). Also, it has been reported that miR-155 augments CD8^+^ T cell antitumor activity in lymphoreplete hosts by enhancing responsiveness to homeostatic cytokines and by causing epigenetic reprogramming of T cell fate (33, 34). Collectively, there is substantial evidence that miRNAs play critical roles in T cell differentiation and function.

In this study, we examined the effects of bortezomib on alterations in miRNA expression in T cells and determined its impact on T cell effector function and exhaustion *in vivo*. Using murine experimental set ups from our previous studies (12–14), we investigated how bortezomib administered intravenously at a low dose of 1 mg/kg of body weight affects miRNA expression in CD8^+^ T cells of mice bearing orthotopic mammary adenocarcinoma that presented a defined low-avidity MHC-I-restricted HA_518-526_ epitope (IYSTVASSL) derived from hemagglutinin (HA) model antigen (35). Compared with the expression in CD8^+^ T cells from naïve tumor-free wild-type mice, bortezomib administration in tumor-bearing mice increased CD8^+^ T cell miR-155 expression concomitant with a decreased expression of downstream immunosuppressive targets, namely, the suppressor of cytokine signaling 1 (SOCS1) and the SH-2 containing inositol 5’-polyphosphatase 1 (SHIP1), inhibitors of JAK/STAT and PI3K/AKT signaling in T cells, respectively. Moreover, activated CD8^+^ T cells treated with bortezomib exhibited a significant reduction in programmed cell death-1 (PD-1) expressing SHIP1^+^ subset of cells. These new data support the previously reported anti-tumor effects of bortezomib in adoptive T cell immunotherapy settings. They underscore a mechanism of action by which bortezomib suppresses PD-1–mediated T cell exhaustion by inducing miR-155–dependent downregulation of SOCS1 and SHIP1 negative regulatory proteins. The findings provide the molecular basis underlying bortezomib-mediated stimulatory effects on antitumor CD8^+^ T cell functions.

## Materials and Methods

### Mice

BALB/c mice at 6–8 weeks old (25–30 g by body weight) were purchased from Harlan (Indianapolis, IN) and used for experiments. Mice were housed in filter-topped cages under specific pathogen-free conditions in Meharry Medical College (MMC) Animal Care Facility and cared for in accordance with the procedures outlined in the National Institutes of Health *Guide for the Care and Use of Laboratory Animals* and Institutional Animal Care and Use Committee (IACUC). MMC is accredited by the Association for Assessment and Accreditation of Laboratory Animal Care International and follows the Public Health Service Policy for the care and use of laboratory animals.

### Tumor and Cell Lines

The murine mammary adenocarcinoma cell line 4T1.2-HA (generated in our laboratory) was maintained in 10% FCS-supplemented standard RPMI-1640 culture medium (Gibco, Invitrogen). The tumor cells were kept at low passage (<5) for experimentation and were regularly authenticated with reference stock to ensure fidelity. Sterility and Mycoplasma testing were also performed regularly. Solid tumors were induced in syngeneic BALB/c wild-type (WT) mice by injecting 2 x 10^6^ 4T1.2-HA cells orthotopically under the mammary pads into the right flank. Following the establishment of palpable tumors for about 14 days (approximate size, 120 mm^3^), mice were injected with therapeutic dose of bortezomib (1 mg/kg body weight) intravenously, as was optimized previously (8). This dose roughly correlates to a transient 15 nM concentration of bortezomib on the basis of the observation that a mouse of 25 g weight has an approximate blood volume of about 1.5 ml. Human lymphoblast T1 cells (174 x CEM.T1) (ATCC^®^ CRL-1991™) were maintained at 37 °C and 5% CO_2_ in 90% Iscove’s modified Dulbecco’s medium (Gibco, Invitrogen) and 10% fetal bovine serum (Gibco, Invitrogen). Cultures were sustained at 1 x 10^6^ cells/ml with medium replenishment every 2 to 3 days. The human HEK-293T cells were maintained in Dulbecco’s modified Eagle’s Eagle/s medium (DMEM) supplemented with 10% (v/v) heat-inactivated fetal bovine serum (FBS) (Gibco, USA), 2 mM glutamine and 1% antibiotics (penicillin–streptomycin) (Gibco, USA), and were cultured at 37°C in a humidified 5% CO_2_ atmosphere.

### Tissue Harvesting and Cell Preparation

Tissues were harvested from mice upon sedation with 2,2,2-Tribromoethanol (Millipore Sigma) and cervical dislocation. Single cell suspensions were prepared from tissue homogenization on the Falcon 40 µm cell strainers in petri dishes containing complete RPMI media. The media containing the cells were transferred to labeled 20 ml conical tubes, then spun down at 2,000 rpm for 5 min at 4°C. Cells were washed twice by aspirating media after centrifugation, resuspending cell pellets in media and centrifuging again. Splenocytes were suspended in 1 ml of ACK buffer (KD Medical, Columbia, MD) for 1 min at room temperature to lyse erythrocytes followed by washing with complete RPMI media and centrifugation. The pellet was resuspended in complete RPMI media, and assessed for viability with trypan blue and cell counts using the Countess (Invitrogen).

### CD8^+^ T Cell Purification and Activation

Lymphocytes were pooled from the spleen and lymph nodes and purified by incubating cells with rat anti-mouse CD8 mAb, followed by positive selection of CD8^+^ T cells with anti-rat IgG microbeads (Miltenyi Biotec). Purity of CD8^+^ T cells was more than 95% as confirmed by flow cytometry. In experiments that involved activation, cells were stimulated with soluble anti-mouse CD3 and CD28 antibodies (1 µg/ml each; Biolegend) for 24, 48, or 72 h.

### *In Silico* Analysis

For the identification of hsa-miR-155 binding sites, the miRNA sequence and the 3’UTR sequence of the target genes were queried on two platforms: RNAhybrid 2.1.2 (36, 37) and RNA Structures-BiFold (38). While RNAhybrid predicts secondary structures between the miRNA and the target mRNA through Minimum Free Energy (MFE) calculations, the RNA Structures-BiFold algorithm considers intramolecular base pairings involved in the secondary structure formation between the two RNA molecules. Conservation of the putative miR-155 binding site across mammalian species was further analyzed by Clustal Omega multiple sequence alignment program (39).

### Transfection of T1 Cells

T1 cells (2x10^5^ cells) grown in 6-well culture plates were transfected with 100 picomols of miR-155 anti-miR or mimic (Dharmacon) or control siRNA (Santa Cruz) using Neon Transfection System (Thermo-Fisher, USA). T1 cells were electroporated using conditions: Voltage-1200 V; Width-40 ms; Pulses-1 and then cultured for 24-36 h, pelleted by centrifugation at 500 g for 10 min and subsequently aliquoted for RNA and protein extraction.

### Immunofluorescence Surface and Intracellular Staining

RBC-depleted splenocytes and/or lymphocytes (1 x 10^6^) were plated in a 96-well U-bottom plate, then spun at 2,000 rpm for 5 min at 4 °C. RPMI medium was flicked off and plate was gently vortexed to break up the pellet. Appropriate dilutions of antibodies were prepared in flow buffer containing 0.5% FBS. 50 µl of a cocktail of fluorochrome-labeled anti-mouse antibodies: CD8-PE, CD8-PerCPCy5.5, CD69-PerCPCy5.5, CD25-PE (BioLegend) were added to the designated wells in a 96-well U-bottom plate following Fc*γ*R-blocking and incubated for 30 min in the dark on ice. After incubation, cells were washed twice with flow buffer with centrifugation at 2,000 rpm for 5 min at 4 °C for washing. Cells were resuspended and fixed in 100 µl of Fix/Perm solution and incubated for 20 min at room temperature. The cells were then washed again using 100 µl of 1X perm/wash and resuspended in 150 ul of 1X perm/wash and placed at 4°C in a dark cover until acquisition and analysis. In some cases, following the surface immunofluorescence staining, to measure intracellular protein levels we used the following intracellular antibodies: SOCS1 (ab62584) and SHIP1 (sc-8425) at 1:200 dilution along with DyLight 488 goat anti-mouse IgG-FITC, DyLight 649 donkey anti-rabbit IgG-APC (BioLegend). Intracellular staining was performed by the incubation of cells at appropriate dilutions in perm/wash buffer for 30 mins at 4°C in the dark. After primary antibody staining, cells were washed twice at 2,000 rpm with 1X perm/wash and again after secondary antibody staining with the same wash protocol and then resuspended in 200 µl of 1X perm/wash for acquisition. For PD1 plus SHIP1 and SOCS1 co-staining, samples followed the protocol described above for intracellular staining, using the following antibodies: anti-mouse PE PD1(135206) and anti-mouse APC CD8α (100712) (Biolegend, dilution 1:100) for surface staining followed by intracellular staining with SOCS1, SHIP (1:200 dilution), goat anti-mouse PerCPCy5.5 IgG (405314) and DyLight 649 donkey anti-rabbit IgG (406404-Biolegend, dilution 1:100).

### RNA Isolation and Quantitative PCR

miRNA or total RNA was extracted using miRNeasy mini kit or RNeasy mini kit (Qiagen) and quantified by reading the optical density at 260 nm. The cDNA was either synthesized using miScript II RT kit (Qiagen) or iScript cDNA synthesis kit (Bio-Rad). Real-time quantification RT-PCR (qRT-PCR) was performed using CFX-96 Real Time System (Bio-Rad). The miScript SYBR Green PCR kit (Qiagen) or iQ SYBR green supermix (Bio-Rad) and gene-specific PCR primers were used in 20-25 µl reactions following protocols recommended by the manufacturer. The conditions used for the miRNA PCR were as follows: 95°C for 15 min, 94°C for 15 s, 55°C for 30 s, 70°C for 30 s (40 cycles). The conditions used for the total RNA PCR were as follows: 95°C for 3 min (one cycle), 95°C for 15 s, 60°C for 45 s (40 cycles), melt curve 55°C to 95°C for 5 s increments. Fold changes in miRNA and total RNA expression were assessed by the ^ΔΔ^CT method. Primer assays and sequences are as follows: Mm_miR-155_1 miScript primer assay (MS00001701), Hs_miR-155_2 miScript Primer Assay (MS00031486), Hs_RNU6-2_11 miScript primer assay (MS00033740).

### Western Blot Analysis

Cell pellets from treated T1 cells were lysed in complete lysis buffer including protease inhibitor. A total of 25 µg of each protein sample was electrophoresed on NuPage 4-12% Bis-Tris gel (Invitrogen) and transferred to nitrocellulose membranes using semi-wet transfer system. The membrane was then blocked in 5% nonfat dry milk in 1X tris-buffered saline-Tween-20 (1X TBST) for 1 h at room temperature with gentle agitation. After blocking, the blots were incubated with specific primary antibodies for SOCS1 (ab62584) at a dilution of 2 µg/ul, SHIP1 (sc-8425) at a dilution of 1:200, and β-actin (ProteinTech 66009-1-Ig) at a dilution of 1:60,000 in 5% milk (in 1X TBST) overnight at 4℃ with gentle agitation. After four 15 min washes each in 1X TBST, blots were incubated with either goat anti-rabbit or goat anti-mouse horseradish peroxidase (Bio-Rad) at a dilution of 1:500 (anti-rabbit, SOCS1), 1:1,000 (anti-mouse, SHIP1), and 1:60,000 (anti-mouse, β-actin) for 1 h at room temperature, with agitation. The blots were washed four times for 15 mins in 1X TBST, and developed by using chemiluminescence reagent and ChemiDoc™ Touch Gel Imaging System (Bio-Rad). The density of each protein band was determined by densitometric analysis using the imageJ software (NIH). Levels of β-actin were determined in each well to verify that equal amounts of protein were loaded. In addition, the density of each protein band was normalized to β-actin to determine relative protein expression to the internal control.

### Luciferase Assay

To determine if *SHIP1* 3’UTR activity is regulated by miR-155, we carried out luciferase reporter assays. pmiReport SHIP1 3’ UTR reporter construct was obtained from Addgene (28). All transfections were performed using the polyethyleneimine (PEI) transfection reagent (40). HEK-293T cells were cultured in 24-well plates overnight and then treated with increasing doses of bortezomib, followed by co-transfection with 100 ng of pmiReporter *SHIP1* 3’ UTR reporter construct and 100 picomols of miR-155 anti-miR. Post-transfection plates were incubated for 24 h. Transfected cells were lysed using 1x GLO lysis buffer (Life Technologies, USA) and luciferase activity was measured using a plate reader (BioTek, USA). Samples were assayed in triplicate and the data are shown as luciferase activity normalized to RFP expression.

### Data Acquisition and Statistical Analysis

Flow data on samples were acquired using the guava EasyCyte 6HT-2L instrument (Millipore), where 100–200 x10^3^ cells were acquired for each sample well. Single color controls were used to determine gating as well as isotype controls were used to ascertain whether there was any non-specific antibody binding. FlowJo 10.6 software (TreeStar) was used to analyze all data. *In silico* analysis for the minimum free energy of the binding between miR-155 and SOCS1/SHIP1 was conducted using RNA Hybrid and Bifold software. Data were combined from each independent experiment and are presented as means ± S.D. or S.E.M. GraphPad Prism 7.0 was used to compare the mean values between groups and statistical significance of differences was determined by using either one-way ANOVA or two-tailed *t*-test with *p* ≤ 0.05 considered statistically significant.

## Results

### Bortezomib Treatment Increases CD8^+^ T Cell miR-155 Expression in Mice With or Without Mammary Tumors

**Figure 1:**
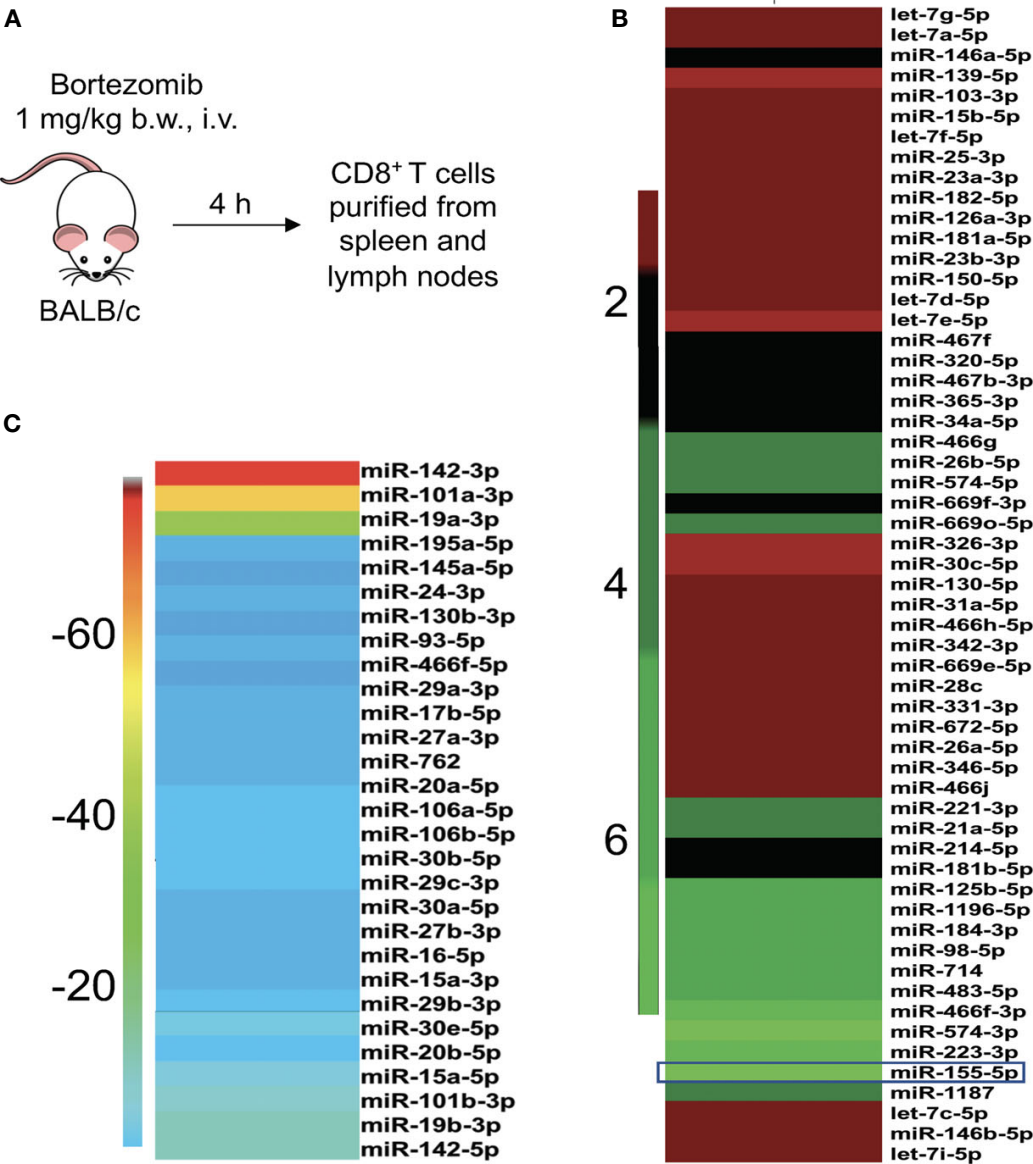
Bortezomib administration in mice affects the expression of miRNAs in T cells. **(A)** BALB/c wild-type mice were treated with bortezomib intravenously (1 mg/kg body weight, approximately 15 nM dose by blood volume). Mice were euthanized 4 h post injection of bortezomib and CD8^+^ T cells were purified from a pool of spleen and lymph nodes. The miScript PCR microarray analysis was performed on miRNA isolated from CD8^+^ T cells. The fold changes of upregulated **(B)** and downregulated **(C)** miRNAs upon bortezomib treatment are depicted by heat plots with a color scale indicated on the sides.

**Figure 2:**
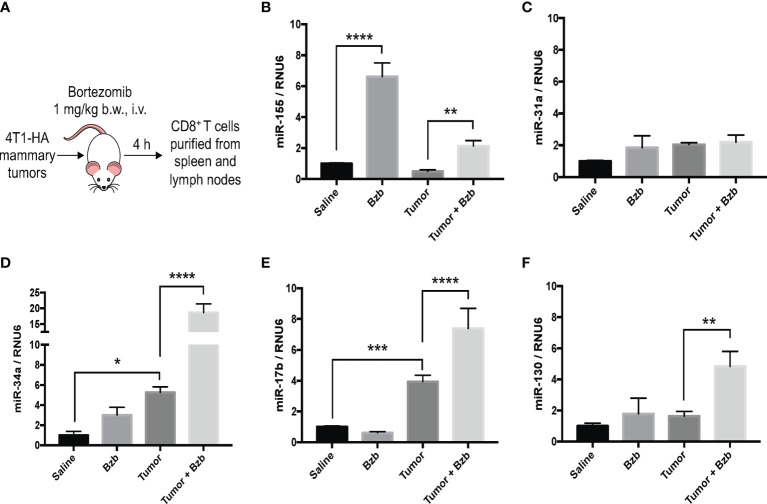
Bortezomib increases CD8^+^ T cell miR-155 expression in mice with or without mammary tumors. Mammary tumors of at least 120 mm^3^ in size were established in a cohort of BALB/c wild-type (WT) mice by orthotopic injection of 2 x 10^6^ 4T1-HA tumor cells into mammary pads. Mice were treated with bortezomib intravenously (1 mg/kg b.w.) and euthanized 4 h post injection. CD8^+^ T cells were purified from a pool of spleen and lymph nodes **(A)**. Expression levels of miR-17b, miR-31a, miR-34a, miR-130, and miR-155 were determined in CD8^+^ T cells by RT-PCR. Values were compared with groups injected with saline **(B–F)**. Data are presented as mean values ± SD from four independent experiments; ^*^ *p* < 0.0066, ^**^ *p* < 0.0021, ^***^ *p* < 0.0003, ^****^ *p* < 0.0001 compared to saline; *n* = 6 per group (ANOVA, one-way).

Gain or loss of function studies have shown that T cells exhibit unique miRNA expression profiles to shape their functions in response to various T cell receptor (TCR) stimuli (20, 41, 42). We have previously reported that bortezomib enhances the antitumor function of T cells in adoptive T cell immunotherapy settings (12–14). Given that miRNAs play key roles in T cell function, we examined whether bortezomib enhances the antitumor function of T cells by regulating cellular miRNAs in T cells. To test this, we administered BALB/c WT mice with bortezomib at 1 mg/kg body weight dose intravenously. This dose mimics the *in vivo* therapeutic regimen established to show maximal antitumor effects in mammary and renal adenocarcinomas (8, 14). Also, assessment of cytokine protein levels in bortezomib-treated naïve mice at this dose showed that expression of the immunostimulatory cytokines IL-2, IL-12p40, IL-12p70, and IL-15 reached peak levels in splenocytes at 4 h after drug administration (12). Thus, we performed a T/B cell miScript PCR array on CD8^+^ T cells purified from a pool of spleen and lymph nodes from mice after 4 h of bortezomib treatment. The microarray analysis showed that bortezomib administration altered the expression of a broad range of miRNAs with several miRNAs being downregulated or upregulated (**Figure 1**). These include the miRNAs such as miR-17b, miR-31a, miR-34a, miR-130, and miR-155 that display a host of functions linked to maintaining T cell activation, proliferation and effector function, and regulating IFN*γ* signaling, exhaustion and memory T cell differentiation (31, 43–46). Specifically, a significant increase in miR-155 expression by 7-fold was observed in CD8^+^ T cells of WT mice that were treated with bortezomib at the therapeutic dose of 1 mg/kg body weight (**Figures 2A, B**). An increase in CD8^+^ T cell miR-155 expression was also noted in tumor-bearing mice following bortezomib administration, although to a lesser degree by about 4.5-fold when compared with CD8^+^ T cells from tumor-bearing mice treated with saline (**Figure 2B**). Bortezomib treatment or tumor growth did not change the expression of CD8^+^ T cell miR-31a expression (**Figure 2C**). Compared to naïve mice without tumors, mice with mammary tumors showed a significant increase in CD8^+^ T cell miR-17b and miR-34a expression, which was further enhanced by bortezomib administration (**Figures 2D, E**). The expression of miR-17b and miR-34a could possibly be exacerbated by tumor growth as bortezomib treatment in mice without tumors did not influence their expression in CD8^+^ T cells. Bortezomib administration also increased CD8^+^ T cell miR-130 expression in tumor-bearing mice (**Figure 2F**).

Taken together, these results suggest that bortezomib administration significantly increased miR-155 expression in CD8^+^ T cells in naïve WT mice. Intriguingly, increased miR-155 expression paralleled the upregulation of immunostimulatory cytokines and effector molecules in these cells following bortezomib treatments (12, 13), implicating miR-155 as a potential candidate driving bortezomib-mediated effects on T cells.

### Suppressor of Cytokine Signaling 1 and SH-2 Containing Inositol Polyphosphate-5-Phosphatase 1 Are Functional Targets of miR-155

**Figure 3:**
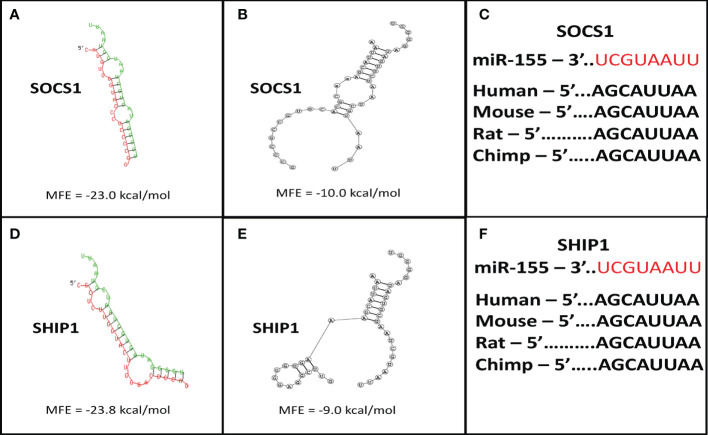
Suppressor of cytokine signaling 1 (SOCS1) and SH-2 containing inositol 5’-polyphosphatase 1 (SHIP1) are targets of miR-155. Target prediction *in silico* analysis was performed for finding the minimum free energy hybridization of a long and a short RNA to predict miR-155 targets. Analysis of the structural binding between miR-155 and SOCS1 or SHIP1 was conducted using the RNAhybrid and Bifold softwares. TargetScan software analysis was also performed to determine whether the binding sequences between miR-155 and SOCS1 or SHIP1 are conserved among multiple species. **(A)** RNA hybrid representative structural binding efficiency and minimum free energy between miR-155 and SOCS1. **(B)** RNA bifold representative structural binding efficiency for the minimum hybrid free energy conformation between miR-155 and SOCS1. **(C)** Target sequence conservation of miR-155 to *SOCS1* 3’ UTR in multiple mammalian species. **(D)** RNA hybrid representative structural binding efficiency and minimum free energy between miR-155 and SHIP1. **(E)** RNA bifold representative structural binding efficiency for the minimum hybrid free energy conformation between miR-155 and SHIP1. **(F)** Target sequence conservation of miR-155 to *SHIP1* 3’ UTR in multiple mammalian species. Values in the quadrants depict minimum free energies.

To examine whether bortezomib-induced increase in miR-155 expression in CD8^+^ T cells is functionally linked to its antitumor functions, we performed molecular target identification studies. First, we employed *in silico* analysis using two independent algorithms, RNAhybrid 2.1.2 (36, 37) and biFold : RNA Structures (38) to enhance prediction accuracy. Both the platforms predicted a miR-155 binding site within the *SOCS1* 3′UTR (**Figure 3A**). Prediction of structural interaction between miR-155 and *SOCS1* target sequence showed the formation of a hairpin-loop structure between the two RNA molecules. The negative minimum free energy calculations suggested a medium to strong genetic interaction between miR-155 and *SOCS1* 3’-UTR (**Figures 3A, B**). Importantly, we also noted that the binding sequence in *SOCS1* 3’-UTR sequences are conserved among several mammalian species (**Figure 3C**), another hallmark of miRNA-mediated post-transcriptional regulation (47). These *in silico* findings are significant since SOCS proteins inhibit cytokine signaling important for T cell survival and function. Specifically, SOCS proteins block the recruitment of the signal transducer and activator of transcription (STAT) proteins to the cytokine receptor, inhibit the activity of Janus kinases (JAK), and target the receptor and JAKs for degradation by the proteasome (48). Therefore, increased expression of miR-155 could block the inhibitory effects of SOCS proteins and increase the antitumor function of T cells.

In addition to *SOCS1*, the *in silico* target scan analysis also predicted a binding site for miR-155 within the 3’UTR of *SHIP1* (**Figures 3D, E**). The structural predictions of miR-155 and *SHIP1* target sequence interaction showed a negative minimum free energy representative of a medium to strong binding between miR-155 and *SHIP1* 3’UTR (**Figures 3D, E**). The binding sequence of miR-155 in the *SHIP1* 3’UTR was also found to be conserved among a number of mammalian species (**Figure 3F**). SHIP1 plays a substantial role in T cell survival and function through the phosphatidylinositol 3-kinase PI3K/Akt pathway. Upon TCR stimulation, PI3K is recruited to the membrane to phosphorylate and convert PI(4,5)P_2_ to PI(3,4,5)P_3._ Proteins such as Akt bind to PI(3,4,5)P_3_ to trigger cytokine signaling and T cell processes. Importantly, SHIP1 removes the 5’ phosphate from PI(3,4,5)P_3_ and inhibit PI3K/Akt signaling (28, 49–52). Therefore, higher levels of miR-155 has the potential to abrogate SHIP1 mediated PI3K/Akt signaling in T cells.

Collectively, these *in silico* observations imply that by inducing miR-155 in T cells bortezomib could drive the downregulation of SOCS1 and SHIP1 proteins. Thus, by intersecting the negative regulators of CD8^+^ T cell function, bortezomib could improve antitumor immunity.

### Bortezomib Treatment Increases the Expression of miR-155 Concurrent With Decreased SH-2 Containing Inositol Polyphosphate-5-Phosphatase 1 Levels

**Figure 4:**
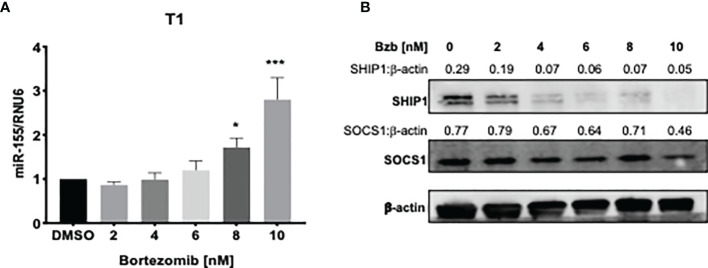
Dose dependent increase of miR-155 expression and decrease of SH-2 containing inositol 5’-polyphosphatase 1 (SHIP1) protein following bortezomib treatment. Human lymphoblast T1 cells were treated *in vitro* for 24 h with increasing concentrations of bortezomib (2, 4, 6, 8, 10 nM). The cells were collected and analyzed for miR-155 expression by RT-PCR and for suppressor of cytokine signaling 1 (SOCS1) and SHIP1 protein levels by Western blotting. **(A)** The expression of miR-155 in T1 cells is shown upon bortezomib treatment at the indicated concentrations. **(B)** SOCS1 and SHIP1 protein levels in T1 cells upon bortezomib treatment at the indicated concentrations. Control samples are treated with DMSO. Numbers above the bands are the indicated ratios of the densitometry values. Data in bar graphs are presented as mean values ± SEM from five independent experiments, each with triplicate values. ^*^ *p* < 0.05, ^***^ *p* < 0.005 compared to DMSO controls (Unpaired *t*-test, two tailed).

We further probed the effects of bortezomib on miR-155 and its targets, SOCS1 and SHIP1, *in vitro* using human lymphoblast T1 cells. Treatment of T1 cells for 24 h with increasing concentrations of bortezomib showed a dose-dependent increase in miR-155 expression (**Figure 4A**). This increase in the expression of miR-155 was most significant in cells treated with 10 nM bortezomib. Interestingly, the increase in miR-155 expression in these cells negatively correlated with SHIP1 protein levels in T1 cells (**Figures 4A, B**). Surprisingly, no significant change in the expression of SOCS1 was observed in bortezomib-treated cells (**Figure 4B**).

**Figure 5:**
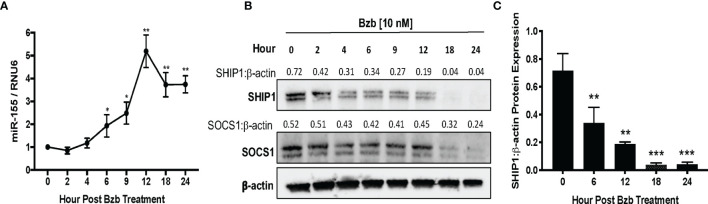
Time kinetics shows the increase of miR-155 and the decrease of its targets suppressor of cytokine signaling 1 (SOCS1) and SH-2 containing inositol 5’-polyphosphatase 1 (SHIP1) in T1 cells following bortezomib treatment. Human lymphoblast T1 cells were treated *in vitro* with 10 nM bortezomib for 2, 4, 6, 9, 12, 18, and 24 h. The cells were collected at each time point and analyzed for miR-155 expression by RT-PCR and for SOCS1 and SHIP1 protein levels by Western blotting. The expression of miR-155 **(A)** and SOCS1 and SHIP1 protein levels **(B)** are shown by function of time in T1 cells upon 10 nM bortezomib treatment. Numbers above the bands are the indicated ratios of the densitometry values. **(C)** Bar graphs depict SHIP1:β-actin ratios for fold changes in SHIP1 protein as calculated from the densitometry values. Data are presented as mean values ± SD from four independent experiments with triplicates; ^*^ *p* < 0.0072, ^**^ *p* < 0.0001, ^***^ *p* < 0.00001 (ANOVA, one-way), compared to 0 h **(A, C)**.

A time kinetics of bortezomib (10 nM) treatment in T1 cells revealed significant changes occurring in the expression of miR-155 beyond 4 h of treatment with a peak expression achieved by 12 h (**Figure 5A**). This was concomitant with a reduction in SHIP1 protein also observed beyond 4 h reaching undetectable levels by 18 h. On the other hand, SOCS1 protein showed a marginal reduction by 18 h that was not statistically significant (**Figure 5B**). Collectively, these data show that bortezomib treatment resulted in an increased miR-155 expression and decreased the protein levels of miR-155 target SHIP1 but not SOCS1. Thus, bortezomib could possibly drive CD8^+^ T cell effector function by modulating miR-155–SHIP1 axis.

### Reduction of miR-155 Expression Abrogates Bortezomib-Mediated Decrease in SH-2 Containing Inositol Polyphosphate-5-Phosphatase 1 Levels

**Figure 6:**
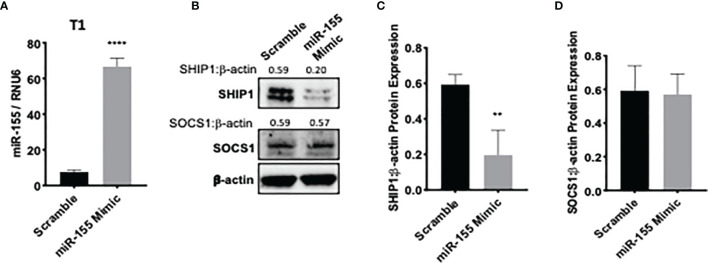
miR-155 overexpression decreases SH-2 containing inositol 5’-polyphosphatase 1 (SHIP1) levels. T1 cells were treated for 24 h with either hsa-miR-155-5p mimic or scramble siRNA plasmids. The expression of miR-155 **(A)** and suppressor of cytokine signaling 1 (SOCS1) and SHIP1 proteins **(B)** are shown in T1 cells 24 h post miR-155 mimic and scramble plasmid transfection. Numbers above the bands are the indicated ratios of the densitometry values. Bar graphs depict SHIP1:β-actin ratios **(C)** or SOCS1:β-actin ratios **(D)** for fold changes in SHIP1 or SOCS1 proteins as calculated from the densitometry values. Data in bar graphs are presented as mean values ± SEM from four independent experiments, each with triplicates. ^**^ *p* < 0.002, ^****^ *p* < 0.0001 for miR-155 mimic *versus* scrambled control (Unpaired *t*-test, two tailed).

To assess whether bortezomib-mediated effects on miR-155–SHIP1 axis is a direct response to bortezomib and not off-target effects, we conducted genetic experiments using miR-155 mimics and inhibitors. T1 cells were treated for 24 h with either hsa-miR-155-5p mimics or scrambled controls. qPCR analysis confirmed a significant increase in miR-155 expression when overexpressed using the miR-155 mimic in comparison to scrambled control (**Figure 6A**). Western blot analysis showed that in cells overexpressing miR-155, expression of SHIP1 protein levels was markedly reduced in comparison to the scrambled control (**Figures 6B–D**). These observations provide further evidence that miR-155 expression is inversely linked to decreased SHIP1 expression.

**Figure 7:**
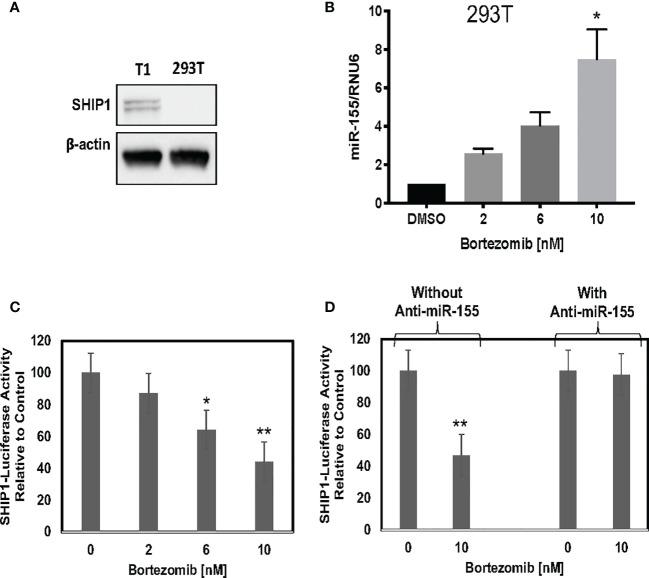
Blockade of miR-155 expression abrogates bortezomib-mediated decrease in SH-2 containing inositol 5’-polyphosphatase 1 (SHIP1) levels. Human HEK-293T cells were treated *in vitro* for 24 h with increasing concentrations of bortezomib (2, 4, 6, 8, 10 nM). The cells were collected and analyzed for miR-155 expression by RT-PCR and for SHIP1 protein level by Western blotting. **(A)** Western blot shows lack of SHIP1 expression in 293T cells in comparison with T1 cells. **(B)** The expression of miR-155 in 293T cells is shown upon bortezomib treatment at the indicated concentrations. 293T cells were transfected with 100 µg of SHIP1 3’-UTR luciferase reporter plasmid and treated with increasing concentrations of bortezomib as indicated in combination with or without 200 pmoles of anti-miR-155. **(C)** Bortezomib’s dose dependent effect on SHIP1-luciferase reporter activity is shown in 293T cells. **(D)** Bortezomib’s effects on SHIP1-luciferase activity in 293T cells in the presence or absence of anti-miR-155 are shown. Data are mean values ± SD from three independent experiments with triplicates; ^*^ *p* < 0.0148 compared to DMSO, ^**^ *p* < 0.0001 compared to DMSO or anti-miR-155 (ANOVA, one-way).

We further assessed a functional link between miR-155 and SHIP1 in bortezomib-treated cells by conducting luciferase-based reporter assay. For this assay, we employed HEK-293T cells as a model since these cells do not express SHIP1 protein endogenously (**Figure 7A**) but show increased expression of miR-155 following bortezomib treatment in a dose-dependent manner (**Figure 7B**). We transfected 293T cells with *SHIP1* 3’-UTR luciferase reporter plasmid and treated the cells with increasing concentrations of bortezomib. Measurement of luciferase activity in the cellular extracts showed that SHIP1 promoter activity is decreased with increasing doses of bortezomib (**Figure 7C**). Moreover, when 293T cells were co-transfected with both the SHIP1 reporter plasmid and anti-miR-155, we observed no change in SHIP1 promoter activity following treatment with 10 nM bortezomib. These results are in clear contrast to the decreased SHIP1 promoter activity in cells without the anti-miR-155 transfection (**Figure 7D**). Thus, results confirm that bortezomib treatment decreases the activity of *SHIP1* 3’UTR by increasing miR-155 expression. Accordingly, the modulation of miR-155–SHIP1 regulatory axis could underlie the increased T cell effector function following bortezomib treatment in tumor-bearing mice (13, 14).

### Bortezomib Treatment Decreases Suppressor Of Cytokine Signaling 1 and SH-2 Containing Inositol Polyphosphate-5-Phosphatase 1 in Activated Primary CD8^+^ T Cells to Diminish Their Exhaustion

**Figure 8:**
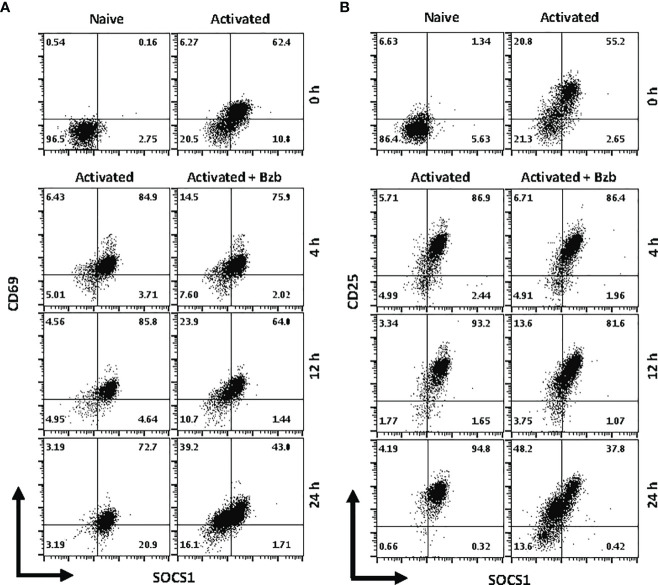
Bortezomib decreases suppressor of cytokine signaling 1 (SOCS1) protein levels in primary activated CD8^+^ T cells. CD8^+^ T cells were purified from the spleen and lymph nodes of naïve BALB/c wild-type (WT) mice. Cells were stimulated *in vitro* with soluble anti-mouse CD3 and CD28 antibodies (1 µg/ml each) for ~20 h followed by treatment with 10 nM bortezomib for 4, 12, and 24 h. With each time point, surface staining for CD8, CD69, and CD25 as well as intracellular staining for SOCS1 and SH-2 containing inositol 5’-polyphosphatase 1 (SHIP1) was performed. Dot plots depict percentages of CD8^+^ cells that express both SOCS1 and CD69 **(A)** or SOCS1 and CD25 **(B)** in the naïve, activated, and activated plus bortezomib-treated groups. Plots are representative of three independent experiments.

**Figure 9:**
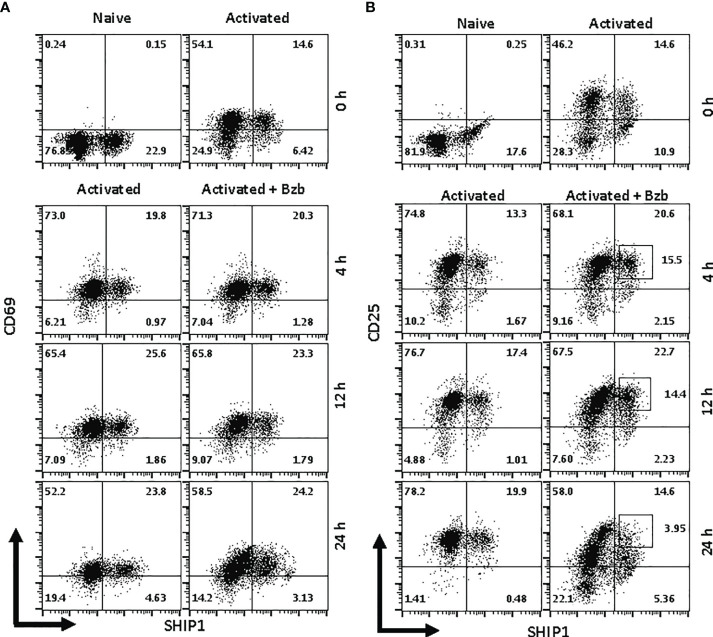
Bortezomib decreases SH-2 containing inositol 5’-polyphosphatase 1 (SHIP1) protein levels in activated CD8^+^ T cells. CD8^+^ T cells were purified from the spleen and lymph nodes of naïve BALB/c wild-type (WT) mice. Cells were stimulated *in vitro* with soluble anti-mouse CD3 and CD28 antibodies (1 µg/ml each) for ~20 h followed by treatment with 10 nM bortezomib for 4, 12, and 24 h. With each time point, surface staining for CD8, CD69, and CD25 as well as intracellular staining for suppressor of cytokine signaling 1 (SOCS1) and SHIP1 was performed. Representative dot plots depict percentages of CD8^+^ cells that express both SHIP1 and CD69 **(A)** or SHIP1 and CD25 **(B)** in the naïve, activated, and activated plus bortezomib-treated groups.

We next determined whether the effects observed in T1 or 293T cells following bortezomib treatment on SOCS1 and SHIP1 proteins were shown by primary CD8^+^ T cells. Purified CD8^+^ T cells from naïve BALB/c wild-type mice were stimulated *in vitro* with anti-CD3 and anti-CD28 antibodies for ~20 h, and then treated with 10 nM bortezomib for another 24 h. We performed intracellular staining for SOCS1 and SHIP1 as well as receptor staining for the early and late T cell activation molecules CD69 and CD25, respectively. At ~20 h of activation CD8^+^ T cells showed higher subsets of CD69^+^SOCS1^+^ (62%) and CD25^+^SOCS1^+^ (55%) phenotypes (**Figure 8**) than CD69^+^SHIP1^+^ or CD25^+^SHIP1^+^ (both 15%) phenotypes (**Figure 9**). This suggested that SOCS1 protein expressed earlier than SHIP1 protein in activated CD8^+^ T cells.

**Figure 10:**
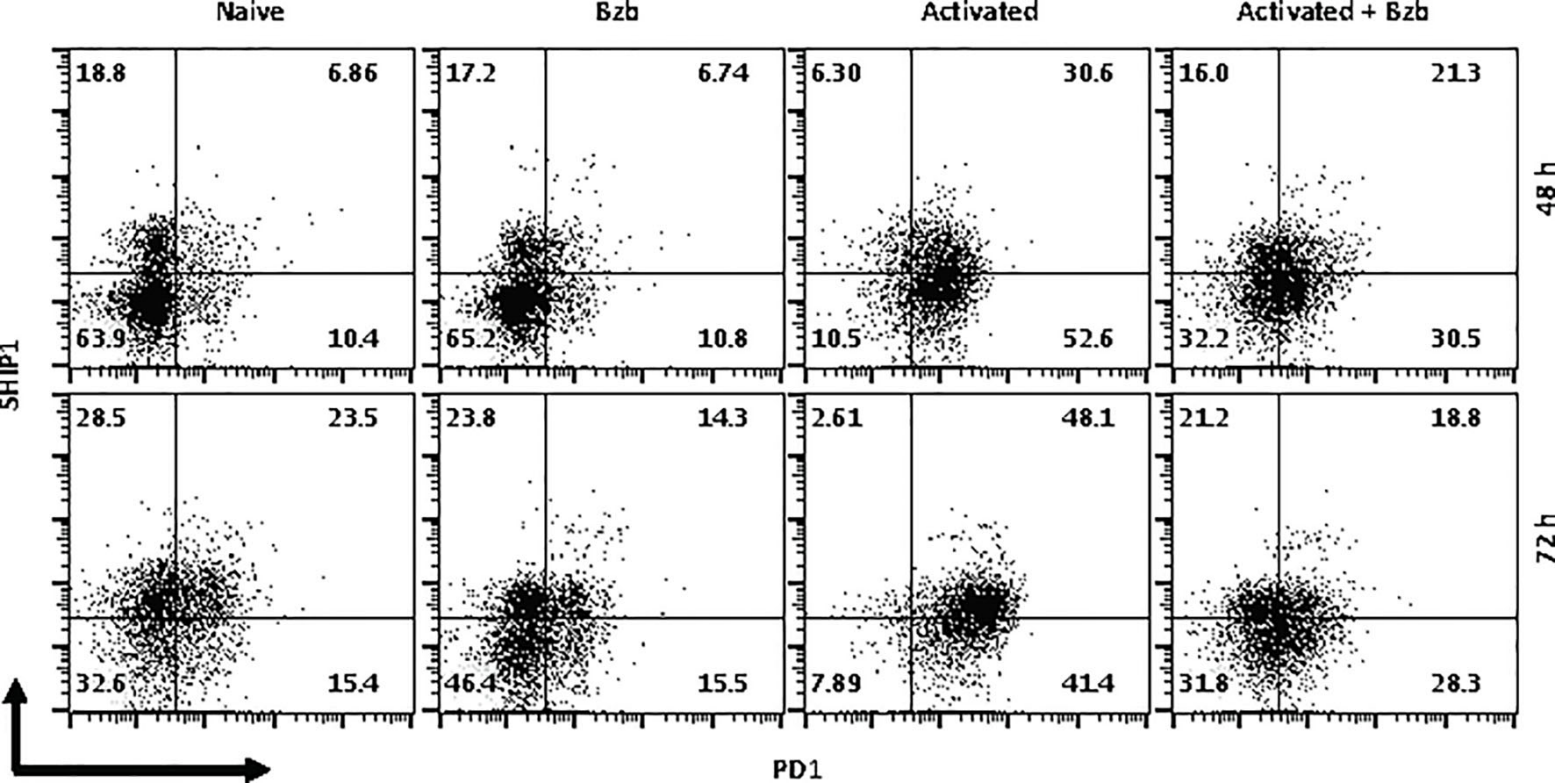
Bortezomib treatment decreases the expression of exhaustion molecule PD1 in activated CD8^+^ T cells. CD8^+^ T cells were purified from the spleen and lymph nodes of naïve BALB/c wild-type (WT) mice. Cells were stimulated *in vitro* with soluble anti-mouse CD3 and CD28 antibodies (1 µg/ml each) for ~20 h followed by treatment with 10 nM bortezomib. Surface staining for CD8 and PD-1 as well as intracellular staining for SH-2 containing inositol 5’-polyphosphatase 1 (SHIP1) was performed after 48 and 72 h post T cell activation. Representative dot plots depict the frequencies of gated CD8^+^ T cell populations expressing SHIP1 and PD-1 in the following groups: naïve CD8^+^ T cells treated with saline, naïve CD8^+^ T cells treated with bortezomib (Bzb), activated CD8^+^ T cells treated with saline and activated CD8^+^ T cells treated with Bzb.

In gated CD8^+^ T cells, CD69^+^SOCS1^+^ cell population decreased from 76% at 4 h post bortezomib treatment to 43% at 24 h (**Figure 8A**). Also, CD25^+^SOCS1^+^ cells decreased from 86% to 37% at 24 h following bortezomib treatment (**Figure 8B**). Similarly, CD25^high^SHIP1^+^ subset of CD8^+^ T cells showed a 74% decrease (16% to 4%) (**Figure 9**). Noticeably, CD25^low^SHIP1^+^ or CD69^+^SHIP1^+^ subsets of CD8^+^ T cells did not show much change following bortezomib treatment (**Figures 9A, B**). Due to the observed differences in kinetics of the expression of SOCS1 and SHIP1 proteins, the effects of bortezomib on SOCS1 protein manifest earlier than SHIP1 protein in primary CD8^+^ T cells. This is distinct from the effects of bortezomib observed on lymphoblast T1 cells, which express constitutively higher levels of activation molecules CD69 (70%) and CD44 (85%) and lack IL-2 receptor α-chain, CD25 (data not shown). Accordingly, in T1 cells bortezomib had a higher impact on SHIP1 levels than SOCS1 whereas in primary CD8^+^ T cells it impacted the expression of both SOCS1 and SHIP1 proteins. This could influence the survival and function of activated CD8^+^ T cells. To explore a possible connection between bortezomib treatment, SHIP1 expression, and T cell exhaustion, we analyzed the intracellular expression of T cell exhaustion molecule programmed cell death-1 (PD-1) on bortezomib-treated activated CD8^+^ T cells. Indeed, at 72 h post activation bortezomib treatment caused a 60% reduction in SHIP1^+^PD1^+^ subset of activated CD8^+^ T cells from 48% to 19% (**Figure 10**).

Thus, depending on the status of cellular activation bortezomib decreases SOCS1 and SHIP1 proteins in CD8^+^ T cells. These data together with the finding that bortezomib treatment decreases the activity of SHIP1 through miR-155 regulation underscore the possible mechanism of action by which bortezomib affects CD8 T cell function. By increasing miR-155 expression bortezomib decreases SOCS1 and SHIP1 negative regulatory proteins leading to an increased PI3K/Akt survival signaling and suppressed PD-1-mediated exhaustion.

## Discussion

Tumor-associated immunosuppression is a major challenge to win the war against cancer. Successful immunotherapies should be able to overcome tumor’s ability to suppress or evade the immune response. Among different strategies of immunotherapies such as CAR-T cells and checkpoint inhibitors, miRNAs are emerging as an attractive target for the development of alternative immunotherapies due to their ability to perform posttranscriptional gene silencing in mammalian cells. Several studies have depicted the role of miRNAs in the development and function of immune cells (15). T cell activation is heavily managed by miRNAs through their ability to alter T cell receptor (TCR) signaling and proliferation (17). Increased expression of miR-155, miR-181a and miR-17 through 92 family of clusters are shown to affect the survival and proliferation of lymphoid and myeloid cells, where miR-155 and miR-181a have been specifically implicated in B and T cell responses. More recently, miRNAs have also been characterized as agents for predicting cancer prognosis, cancer therapy, and cancer biomarkers (53–55).

Bortezomib is a dipeptidyl boronate that functions to inhibit the ubiquitin-proteasome pathway responsible for protein turnover. Bortezomib was approved by the FDA as the first therapeutic proteasome inhibitor for the treatment of multiple myeloma and mantle cell lymphoma (4–7). Subsequently, bortezomib showed efficacy in treating relapsed or refractory myeloma (10) and solid tumors such as advanced stage non-small cell lung-cancer (11). Also, bortezomib treatment in mice influenced tumor microenvironment by increasing the levels of immunostimulatory cytokines IL-2, IL-12 and IL-15 (12) and enhanced the production of IFN*γ* and expression of effector molecules perforin, granzyme B and FasL in CD8^+^ T cells (13, 14). These effects improved adoptive T cell therapy against cancer by predominantly enhancing FasL–mediated CD8^+^ T cell cytotoxicity (14). However, the mechanisms by which bortezomib brought about these T cell intrinsic effects remained unclear.

Results of this study show that bortezomib administered at a 1 mg/kg body weight dose in WT BALB/c mice modulates the expression of a broad range of miRNAs, some of which are involved in T cell function. Specifically, in mice bearing mammary adenocarcinomas bortezomib treatment augmented the expression of miR-17b, miR-34a, miR-130 and miR-155. These miRNAs are linked to T cell effector function, exhaustion and memory differentiation (31, 43–46). While bortezomib treatment or tumor growth did not change the expression of CD8^+^ T cell miR-31a expression, tumor growth exacerbated CD8^+^ T cell miR-17b and miR-34a expression, which was further enhanced by bortezomib treatment. The most striking increase was, however, observed in miR-155 expression in CD8^+^ T cells of mice with or without tumors following bortezomib administration.

It has been suggested that miR-155 can increase cytokine signaling in CD8^+^ T cells by targeting SOCS1 pathway as miR-155^-/-^ CD8^+^ T cells display reduced physiological levels of pSTAT5, resulting in decreased proliferation and survival due to limited IL-2 cytokine signaling (29, 56). SOCS proteins inhibit cytokine signaling by a number of ways, including blocking the recruitment of STAT proteins to the cytokine receptor, or targeting the receptor or JAKs for degradation by the proteasome (48). Another signaling pathway that connects to miR-155 in T cells is the PI3K/Akt pathway. The stimulation of TCR recruits PI3K to the membrane where they phosphorylate and convert PI(4,5)P_2_ to PI(3,4,5)P_3._ Akt protein binds to PI(3,4,5)P_3_ and triggers cytokine signaling and T cell activation, proliferation and survival. SHIP1 functions to remove the 5’ phosphate from PI(3,4,5)P_3_ inhibiting PI3K/Akt signaling in T cells (28, 49–52). SHIP1 also inhibits the T-box transcription factor T-bet, a transcriptional regulator of IFN-*γ* production (31, 57–59). Studies have indicated defective expression of IFN*γ* in miR-155^-/-^ CD4^+^ T cells (31, 60). In addition, increased levels of SHIP1 and SOCS1 in miR-155^-/-^ T cells are known to inhibit NF-κB activation (31, 61–63). Reciprocally, NF-κB signaling stimulates the expression of miR-155 in T cells causing it to target SHIP1 and induce IFN-*γ* production (58). Indeed, the *in silico* target scan analysis predicted structural interaction between miR-155 and *SOCS1* target sequence due to the formation of a hairpin-loop structure between the two RNA molecules. The negative minimum free energy calculations suggested a medium to strong genetic interaction between miR-155 and *SOCS1* 3’-UTR. Moreover, the binding sequence of miR-155 within the 3’ UTR of *SOCS1* and *SHIP1* are conserved among various species. These findings suggest that by inducing miR-155 expression bortezomib could downregulate SOCS1 and SHIP1 proteins, the negative regulators of T cell function. In addition, miR-155 has been shown to directly target SOCS1 in dendritic cells (DC) altering the production of IL-12p70 in DCs (64–66). Furthermore, NF-κB signaling stimulates the expression of miR-155 causing it to target SHIP1 and induce IFN-*γ* production (58).

Treatment of human 293T and lymphoblast T1 cells *in vitro* for 24 h with increasing concentrations of bortezomib showed a dose-dependent increase in miR-155. The increase in the expression of miR-155 was most significant at 10 nM bortezomib concentration. A time kinetics of bortezomib (10 nM) treatment in T1 cells revealed significant changes occurring in the expression of miR-155 beyond 4 h of treatment with a peak expression achieved by 12 h. This was concomitant with a reduction in SHIP1 protein also observed beyond 4 h reaching undetectable levels by 18 h. On the other hand, SOCS1 protein showed a marginal reduction around 18 h that was not statistically significant. Thus, bortezomib decreases the protein levels of miR-155 targets inversely to that of miR-155 expression, with a profound effect on SHIP1 protein levels. Bortezomib could possibly drive CD8^+^ T cell effector function by modulating miR-155-SHIP1 axis.

Genetic experiments using miR-155 mimic and inhibitors confirmed the connection between bortezomib treatment and miR-155’s expression and the effects observed on its targets. Cells overexpressing miR-155 showed a remarkable decrease in SHIP1 but not in SOCS1 protein levels in comparison to the scramble control. This suggests that miR-155 may play an active role in decreasing SHIP1 expression. A functional link between miR-155 and SHIP1 was demonstrated by the diminished *SHIP1* promoter activity with increasing doses of bortezomib. Conversely, bortezomib had no effect on SHIP1 luciferase reporter activity in the cells that were given the miR-155 inhibitor, further establishing that bortezomib drives SHIP1 activity or lack thereof by increasing miR-155 expression. These findings suggest that the modulation of miR-155–SHIP1 axis could underlie the increased T cell effector function following bortezomib treatment in tumor-bearing mice (13, 14). Also, miR-155 regulates dendritic cell (DC) activity as well as cytokine production by directly targeting SOCS1 and altering the production of IL-12p70, thereby priming antitumor and antiviral immune responses (64–66). Further, NF-κB signaling stimulates the expression of miR-155 causing it to target SHIP1 and induce IFN-*γ* production (58).

In primary CD8^+^ T cells, bortezomib treatment impacted their exhaustion by decreasing CD69^+^SOCS1^+^ cell population by 43% and CD25^+^SOCS1^+^ cells by 56% at 24 h. Likewise, CD25^high^SHIP1^+^ subset of CD8^+^ T cells showed a 74% decrease albeit in a smaller proportion of cells (16%). Strikingly, CD25^low^SHIP1^+^ or CD69^+^SHIP1^+^ subsets of CD8^+^ T cells did not show much change following bortezomib treatment. This suggested an increased survival of activated CD8^+^ T cells as confirmed by a 60% reduction in SHIP1^+^PD-1^+^ subset of activated CD8^+^ T cells.

Altogether, the results link miR-155 to SOCS1 and SHIP1 expression in CD8^+^ T cells. Both SOCS1 and SHIP1 are negative regulators of PI3K/Akt/STAT signaling (28, 48–52). Our previous studies have shown that bortezomib increases phosphorylation of STAT5 and Akt in T cells (12), which could be a result of the inhibition of SOCS1 and SHIP1 due to bortezomib’s stimulatory effects on miR-155 expression. Bortezomib-induced increase in eomesodermin and T-bet expression leading to an increase in the expression of IFN*γ*, perforin, and granzyme B (13) possibly links T-bet regulation to an increased PI3K/Akt/STAT5 signaling and a decreased PD-1 expression in CD8^+^ T cells (40, 59, 60, 67–70). The transcription factor T-bet plays a major role in the generation of T cell effector function in conjunction with miR-155 expression. Studies show that miR-155 modulates T-bet levels in direct correlation with the SHIP1 target gene; SHIP1^-/-^ CD8^+^ T cells express 61% more T-bet than WT CD8^+^ T cells (59, 60). The mechanism as to how SHIP1 negatively regulates T-bet is associated with mTOR signaling, which is downstream of PI3K signaling. SHIP1 inhibits PI3K signaling leading to a reduction in mTOR signaling and ultimately decreasing T-bet. Likewise, the ability of miR-155 to suppress SHIP1 could lead to an amplification of PI3K and mTOR signaling, thus increasing T-bet (40, 59, 67, 68). Interestingly, T-bet directly represses the transcription of the gene that encodes for the immune cell exhaustion marker, PD-1 as evident by the increased PD-1 levels in T-bet^-/-^ mice (69, 70).

**Figure 11:**
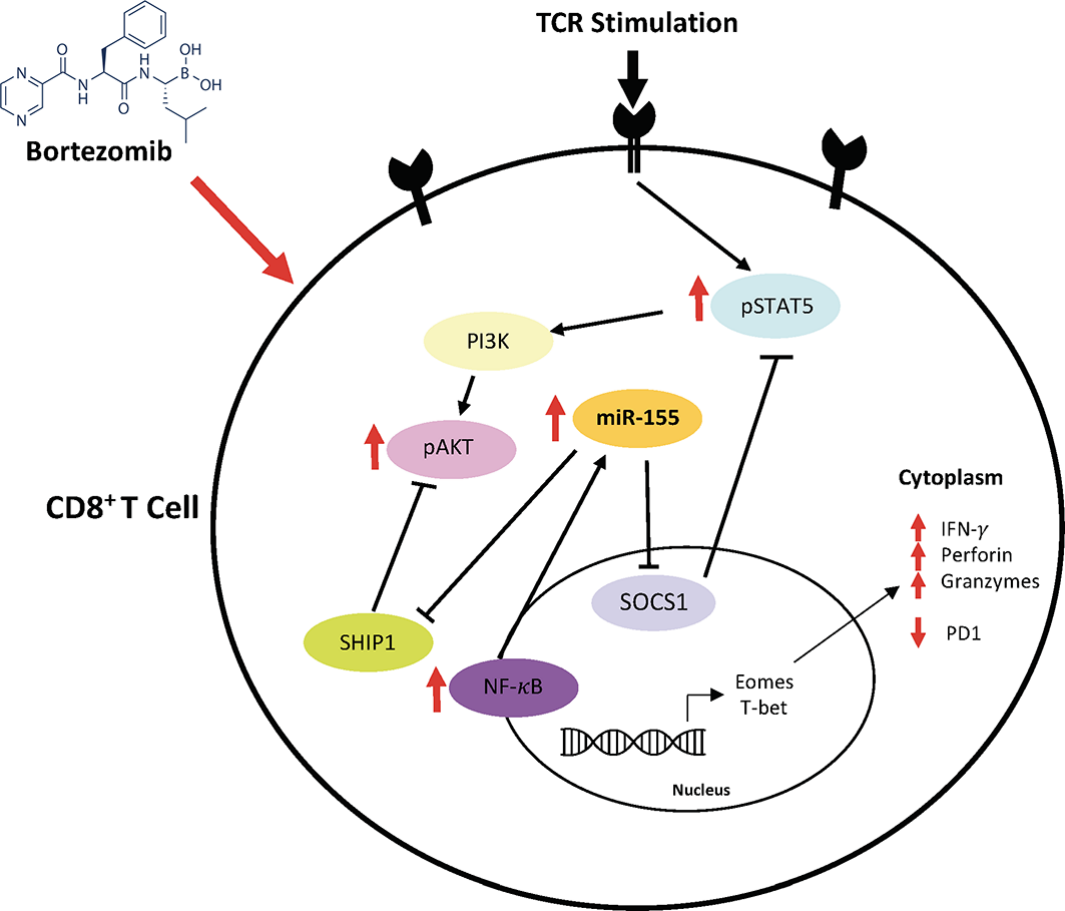
Bortezomib-mediated miR-155 and NF-κB signaling crosstalk to increase CD8^+^ T cell effector function. Bortezomib enhances crosstalk between the miR-155–SOCS1/SHIP1–T-bet–PD-1 axis and NF-κB signaling in CD8^+^ T cells. Bortezomib treatment increases NF-κB activity and miR-155 expression, thereby causing a decrease in its inhibitory targets SHIP1 and SOCS1. The inhibition of these targets in turn lead to decreased PD-1-mediated exhaustion and increased expression of effector molecules in CD8^+^ T cells.

Based on these findings, a possible mechanism of action mediated by bortezomib emerges as illustrated in **Figure 11**. Bortezomib increases NF-κB activity (13), which likely plays a role in increasing miR-155 expression. It is noteworthy that bortezomib sustains increased expression of T cell activation markers CD44 and CD25, improving the production of IFN-*γ*, T-bet, eomesodermin, perforin, and granzyme B allowing for amplified antitumor cytolytic function (13, 14). Present results suggest a bortezomib-mediated crosstalk between the miR-155–SOCS1/SHIP1–T-bet–PD-1 axis and NF-κB signaling stemming from bortezomib’s positive effects on increasing the phosphorylation of IκB kinase, IκBα and p65 (13). Additionally, bortezomib increased the phosphorylation of mitogen-activated protein kinase p38, Akt, and STAT5 in tumor infiltrating CD8^+^ T cells, uncovering a connection between PI3K/Akt/NF-κB/STAT5 pathways (12). Collectively, these studies underscore the profound effect bortezomib has on CD8^+^ T cell function in the context of activation and cytotoxic activity in the tumor microenvironment.

Thus, bortezomib promotes the signaling axis of miR-155–SOCS1/SHIP1–T-bet–PD-1 that enhances T cell survival cytokine signaling and delays exhaustion. This could explain the improved antitumor effector function CD8^+^ T cells observed in the presence of bortezomib (12–14). These results support the approach that bortezomib combined with other immunotherapies would lead to improved clinical outcomes by overcoming tumor-induced exhaustion or tolerance in CD8^+^T cells.

## Data Availability Statement

The raw data supporting the conclusions of this article will be made available by the authors, without undue reservation.

## Ethics Statement

The animal study was reviewed and approved by Meharry IACUC.

## Author Contributions

AS conceived and designed the study. AR prepared the first draft. AR and MT generated data for **Figures 1** and **2**, AR, EC, JP, and CD for **Figures 3**–**7**, and MA and AS for **Figures 8**–**10**. AR, CV, and AS designed final figures. All authors contributed to the article and approved the submitted version.

## Funding

This work was supported by funds to AS from the following National Institutes of Health (NIH) grants: U54 CA163069, U54 MD007593, SC1 CA182843, SC1 CA182843-07S1, and R01 CA175370. AR is supported by the NIH training grant T32 5T32HL007737. This work was also partly supported by the NIH grants R01 AI136740, R56 AI122960, R24 DA036420, and U54 MD007593 to CD and U54 MD007593 to JP. The authors have no other relevant affiliations or financial involvement with any organization or entity with a financial interest in or financial conflict with the subject matter or materials discussed in the manuscript apart from those disclosed. There was no role of the funding bodies in the design or writing of the manuscript.

## Conflict of Interest

The authors declare that the research was conducted in the absence of any commercial or financial relationships that could be construed as a potential conflict of interest.
